# Extracurricular Humanism in Medicine Initiative and Medical Student Wellness: Retrospective Study

**DOI:** 10.2196/37252

**Published:** 2022-09-16

**Authors:** Elizabeth Diane Auckley, Jeff Barbee, Nicole Verbeck, Tracie McCambridge, Linda Stone, Jennifer Garvin

**Affiliations:** 1 The Ohio State University College of Medicine Columbus, OH United States; 2 Office of Curriculum and Scholarship The Ohio State University College of Medicine Columbus, OH United States; 3 Department of Medical Education The Ohio State University College of Medicine Columbus, OH United States; 4 Health Information Management and Systems Divisions Ohio State University Columbus, OH United States

**Keywords:** humanism, extracurricular, stress, burnout, medical student, student, academic success, wellness

## Abstract

**Background:**

Humanism in Medicine Initiative (HIMI), an extracurricular program at Ohio State University College of Medicine (OSUCOM) with 27 subgroups, fosters the humanities. Stress and burnout among first- and second-year medical students are prevalent across the United States. Solutions for stress among first- and second-year medical students have been proposed, but no gold standard exists. The relationship of humanism with stress and burnout has yet to be described in the literature.

**Objective:**

This study investigates the relationship between participation in the HIMI and stress, burnout, and academic success among first- and second-year medical students.

**Methods:**

First- and second-year medical students enrolled at OSUCOM between August 2018 and August 2019 were recruited. Attendance in the HIMI and membership records were used to measure their participation. Curricular examination scores and those on Step 1 of United States Medical Licensing Examination (USMLE) were used to measure academic success. Stress and burnout were measured using the Maslach Burnout Inventory and the Perceived Stress Scale.

**Results:**

In total, 412 students were enrolled with 362 (87%) students participating in HIMI. Those with high participation were more often Black, Asian, female, or with a humanities undergraduate major compared to the overall study population. There were significant relationships between Gold Humanism Honors Society (GHHS) induction and participation of first- and second-year medical students in service- (*χ*^2^_1_*=*5.8, *P<*.05) or leadership-focused (*χ^2^*_1_*=*19.3, *P<*.001) HIMI groups. Medium levels of participation in the HIMI were associated with significantly higher stress. Performance on the Step 1 USMLE was not significantly associated with participation levels in the HIMI (low=233.7 vs high=238.0; *P*=.10).

**Conclusions:**

The HIMI is an extracurricular program vastly utilized by first- and second-year medical students at OSUCOM and did not impact Step 1 USMLE scores. Medium participation in the HIMI was associated with higher stress, and service- and leadership-focused HIMI participation was associated with a higher level of induction to the GHHS. This study identifies areas for future studies to understand the relationship of the HIMI with stress and academic success.

## Introduction

Stress and burnout are well known to the majority of medical students in the United States [1-5]. Stress was first described as the “nonspecific response of the body to any demand made upon it” [6] but has since been recognized as a potentially negative state of being, where the body is threatened by conditions that create imbalance and endanger homeostasis [7]. Burnout, initially described as excessive burdens of energy, strength, or resources in work environments, resulting in malaise, fatigue, cynicism, frustration, and inefficacy [8], is now well understood as a syndrome of emotional exhaustion, depersonalization, and decreased personal achievement due to human interaction at the workplace [2,9]. Stress and burnout often embody a cyclical, perpetuating relationship [2,10] that preclinical medical students are uniquely vulnerable to experiencing [1,10]. In the already hypercompetitive medical school environment with frequent high-stakes formal assessments, students cite unanticipated informal verbal quizzing, feeling useless, and depersonalized preclinical medical education as specific sources of stress and burnout in addition to sources from life outside of school [1,11]. Both psychological conditions are associated with psychological morbidity, anxiety and depression, alcohol and drug abuse, suicidal ideation, and suicide in medical students [1,12,13].

Despite being recognized as a prevalent issue among medical students for at least 4 decades [14,15], no gold standard of stress and burnout reduction for this population has been described [12]. The focus of improvement has shifted from treating impacted students individually to changing the environment of preclinical medical school to be less stressful overall [15]. However, stress and burnout solutions for medical students remain under investigated, with few studies including empirical data for specific interventions [12]. Intervention in medical school is critical, as these psychological conditions in physicians have been found to originate from early-career training in medical school [14]. Further, physicians with resilience, an important phenomenon to fight stress and burnout, are more prepared to create successful patient-physician relationships because they are better able to provide hope and empathy to patients [16].

While stress and burnout are increasingly becoming an important consideration of medical education reforms, humanism has also gained the attention of medical educators in recent decades. The Arnold P. Gold Foundation defines a humanistic health care practitioner as one with integrity, excellence, collaboration and compassion, altruism, respect and resilience, empathy, and service [17]. The Arnold P. Gold Foundation recognizes humanism in medical students through the national honors society induction for the Gold Humanism Honors Society (GHHS). The GHHS selects members based on peer nominations and individual applications for those with strong nominations. Induction into GHHS at OSCOM requires strong peer nominations during the third year of medical school, followed by an invitation to complete an essay-based application. GHHS members select new inductees after rigorous review of deidentified applications and peer nominations. GHHS induction is intended to be impartial. Local chapters of GHHS are limited to inducting a maximum of 15% of each class. Humanism is often considered the foundation for professionalism, which is defined as a way of acting to meet the expectations of the workplace. Professionalism only becomes authentic when humanism, the morals and values that guide ones obligation to serve others especially when in need, is fostered [18]. Calls to protect innate humanism in students [19], foster growth of humanism in medical school culture [18,20,21], and add humanism to the top 4 goals of health care [22] are supported by staff and students alike [23], but specific initiatives and measurement of humanism has been difficult to describe [18-20,24]. Further, the impact of humanities and culture of humanism on preclinical medical students’ stress and burnout has not been previously reported, based on our review of the literature.

This study investigates the impact of first- and second-year medical students’ engagement with HIMI on their stress, burnout, and academic success. HIMI, a portion of the Linda C. Stone MD Program for Humanism and the Arts in Medicine, is a voluntary extracurricular program at the Ohio State University College of Medicine (OSUCOM), which has been developed and is led by students with the goal of fostering a culture of humanism among first- and second-year medical students. Begun in 2009, it now embodies 27 unique student organizations that allow students to engage in the arts and humanities with their peers. Many isolated aspects of HIMI have been supported as general stress- and burnout-reducing activities for students in the literature, including animal therapy [25-27], music [5], peer mentor programs [16,28,29], emotional expression with peers [29-32], student retreats [29], visual arts [33], and being student-led [13].

## Methods

### Ethical Approval

This study was approved by the institutional review board at the Ohio State University (IRB# 2020B0173) and approved for waived consent.

### Recruitment

The setting for this study was a single-center retrospective study conducted at OSUCOM (Columbus, Ohio), a large, Midwestern medical school. The medical school curriculum is structured as two years of lectures and small group-based classes with bimonthly clinical experiences. The third year involves clinical rotations with weekly case discussions, and the fourth year requires specialty specific rotations, an emergency medicine clinical rotation, and an advanced elective. Within this curriculum, Humanism is one option for fourth-year elective courses, and a Humanism in Medicine extracurricular group exists to supplement medical student experiences on an optional basis. This study involved medical students enrolled at OSUCOM from August 2018 to August 2019. All first- and second-year [34] medical students were eligible and enrolled in the study unless they opted out. Students were given the option to terminate participation by opting out, through an email notification. Student demographic information was obtained from internal admissions records for those enrolled.

### Participation and Leadership in the HIMI

First- and second-year medical student participation in the HIMI is defined as attending any event hosted by a student group in the HIMI, organizing an event, leading a group or event, or nominating a peer for a nomination-based group during August 2018 to July 2019. Participation was measured by internal HIMI records of attendance, membership, and leadership. Any amount of participation in a single HIMI group qualified for participation for that group, and the total number of unique HIMI groups participated in was measured for each student. In 2018-2019, HIMI encompassed 27 unique student groups, categorized as art-focused (eg, Dance in Medicine and Photography/Film,), service-focused (eg, Life Support Group and Peer Mentoring), or leadership-focused (eg, Medicine and the Arts Board and Servant Leadership). Student leaders, who had been nominated or recognized by the HIMI as a student organizer of the group, were also recorded.

### Academic Outcomes

Academic success was measured on the basis of the Step 1 United States Medical Licensing Examination (USMLE) score and student Objective Structured Clinical Examination (OSCE) scores. The OSCEs have been administered at most medical schools for more than 4 decades [35] with the goal of measuring communication skills and clinical tasks through directed interactions with several standardized patients [36]. OSCE scores are reported as percentages (0%-100%), and the last OSCE score for the 2018-2019 school year was used for analysis. Step 1 is the first of 3 examinations in the USMLE series. This is a nationally standardized examination required for medical school graduation and licensing that examines student understanding of basic biomedical principles and clinical applications. Step 1 is administered after the completion of didactic coursework and is taken after the second year at OSUCOM [37]. Scores are reported numerically out of 300 and correspond to percentiles nationally [38]. OSCE and Step 1 USMLE scores were obtained from the Office of Curriculum and Scholarship at OSUCOM.

### Stress and Burnout

Stress and burnout were measured using the Maslach Burnout Inventory (MBI), Perceived Stress Scale (PSS), Perceived Cohesion Scale (PCS) [39], and quality of life (QOL) measure. These surveys were administered independently by OSUCOM in a voluntary survey to students in spring 2018. The MBI is based on a 7-point scale of frequency from “never” to “daily” responses to 22 statements organized in 3 sections corresponding to the 3 parts of burnout: emotional exhaustion (9 statements), depersonalization (5 statements), and reduced personal accomplishment (8 statements) [9]. The MBI’s items are derived from the 1996 Maslach Burnout Inventory Manual. The MBI has been validated as a measure of burnout in preclinical medical students [40]. The PSS is a measure of how one’s current life circumstances are interpreted as stressful [41]. A 10-item form of the Likert-scale–based self-report PSS instrument was used, and scores were reported between 10 and 50, with lower scores indicating lower stress. The PSS is validated for populations with at least junior high school education and has been reported to have an internal consistency of 0.85 [42]. The PCS measures sense of belonging and morale within a population and records a response from 0=“strongly disagree” to 10=“strongly agree” to 6 statements, 3 pertaining to sense of belonging and 3 to morale [43]. The population noted in the statements was “medical school class” and “medical school” in this survey. A revised, Bollen and Hoyle form of the Likert-scale self-report PCS [43] was used, which reported scores between 1 and 60, and lower scores indicated less perceived cohesion. QOL was measured using a linear analogue self-assessment, which scores QOL as 1-5, with 1=“as bad as it can be” and 5=“as good as it can be”; this QOL measure has been validated in a wide set of populations [44].

### GHHS Induction

Induction into the GHHS was recorded from internal records of the GHHS. The GHHS was established by the Arnold P. Gold Foundation in 2002 and recognizes students who embody humanistic qualities such as empathy and integrity and serve as role models and advocates for humanism in medicine. The GHHS has been added as a qualification on the Electronic Residency Application Service for students to identify themselves as members, suggesting its importance [45].

### Statistical Analysis

To understand the potential relationship between participating in an HIMI group (ie, arts, leadership-, or service-focused organization) and induction into the GHHS, second-year students, who were eligible to be inducted into GHSS the following year, were grouped dichotomously as having participated in at least one activity, or not at all. A chi-square goodness-of-fit test was conducted for each category of the HIMI group to determine if the proportions of students who participated in at least one group and inducted into the GHHS was similar to those of students who did not participate in any HIMI groups.

Examining the HIMI program and wellness constructs such as burnout and perceived stress, student participation was demarcated into 3 levels of participation: low (0-1 groups), medium (2-3 groups), and high (4 or more groups). This grouping was performed to better differentiate potential effects and attempt to obtain evenly balanced groups. ANOVA was performed to identify any significant differences. All assumptions were checked to ensure this was the appropriate analysis technique.

When studying the perceptions of belonging and morale from a cohort and school viewpoint, the limited data required the participation variable to be collapsed to a dichotomous grouping. The low participation group attended 1 or 0 HIMI groups, while the high participation group attended ≥2 groups. Owing to failure of the assumption of normality, the nonparametric Kruskal-Wallis test was applied. These survey instruments were optional for students to complete and required additional consent for research.

Dichotomous grouping into low and high group participation groups was also used to test for significant relationships with the Step 1 USMLE scores of second-year students. An independent samples *t* test was performed for this analysis. This analytical approach was continued to examine the Professionalism OSCEs during the last second-year evaluation.

The final relationship studied was between participation in the HIMI and the Interpersonal-Communication Skills OSCE scores of first-year students. This OSCE was conducted during the Endocrine-Reproduction block. These data allowed for the use of the trichotomous grouping of low, middle, and high participation levels. ANOVA was performed for this analysis with no assumption violations. All statistical analyses were performed using RStudio (version 1.3.959; RStudio Team).

## Results

### Recruitment and Demographics

**Figure 1:**
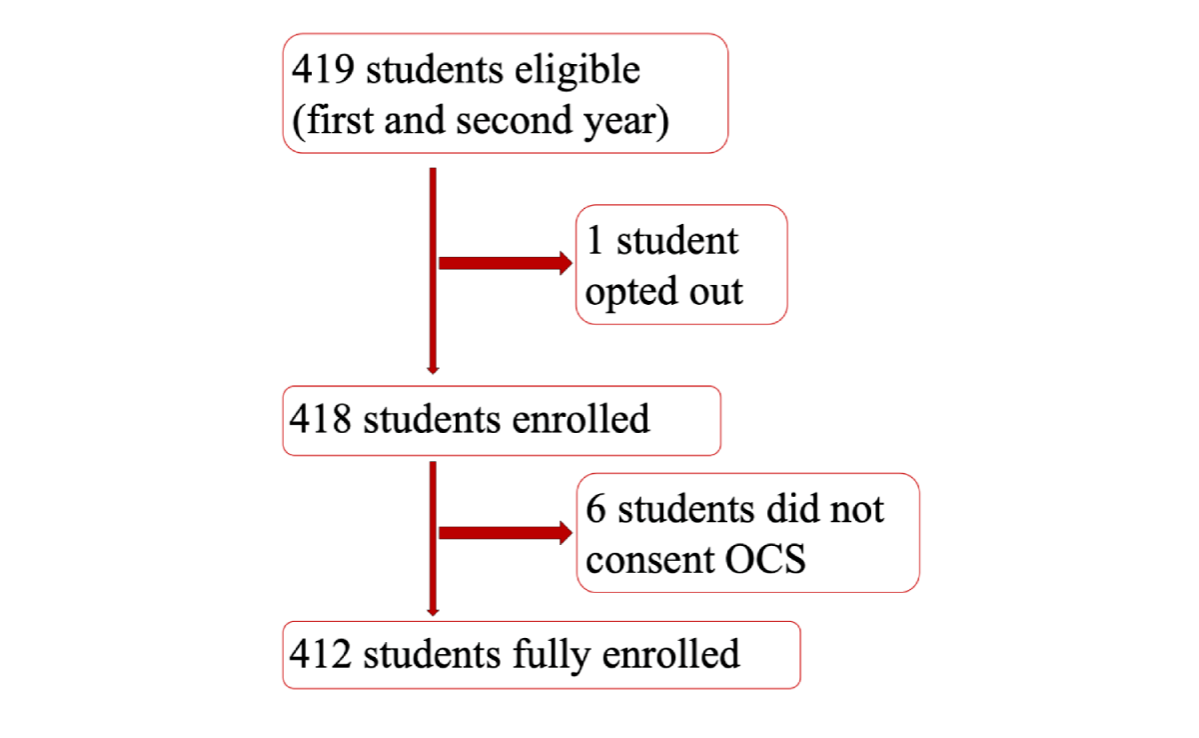
Recruitment of participants. There were 419 first- and second-year students enrolled in Ohio State University College of Medicine (OSUCOM) in 2018-2019. After an email invitation to opt out of the study, 1 student opted out of the study. Six students did not provide consent to the Office of Curriculum and Scholarship (OCS) for the use of demographic and academic data. The analysis included the remaining 412 students. Surveys were optional for students and some data were incomplete.

In total, 419 first- and second-year students were enrolled at OSUCOM during the study period, and 412 met inclusion criteria for analysis (Figure 1). Regarding race and ethnicity, the study population comprised 48.8% White (n=201), 8.5% Black (n=35), and 19.9% Asian (n=82) students, and 22.8% of participants did not respond (n=94), and regarding gender, the study population comprised 45.4% females (n=188) and 41.7% males (n=172), and 12.8% of participants did not respond (n=52). Most participants did not have a humanities undergraduate major (n=338, 82.0%) as opposed to 8.4% (n=35) of participants who had a humanities major, and 9.6% (n=39) of participants did not respond (Table 1).

**Table 1:** Demographic information regarding participants in the Humanism in Medicine Initiative (HIMI) between 2018-2019 (N=412).

			Low participation in the HIMI (n=180), n (%)		Medium participation in the HIMI (n=147), n (%)		High participation in the HIMI (n=85), n (%)		Total, n (%)
**Race**									
	Asian	30 (16.7)		30 (20.4)		22 (25.8)		82 (19.9)	
	Black	19 (10.6)		8 (5.4)		8 (9.4)		35 (8.5)	
	White	86 (47.8)		80 (54.4)		35 (41.2)		201 (48.8)	
	No response	45 (24.9)		29 (19.8)		20 (23.6)		94 (22.8)	
**Gender**									
	Female	59 (32.7)		75 (50.7)		54 (63.5)		188 (45.4)	
	Male	90 (50.0)		61 (41.5)		21 (24.7)		172 (41.7)	
	No response	31 (17.3)		11 (7.8)		10 (11.8)		52 (12.8)	
**Undergraduate major**									
	Humanities	11 (6.1)		16 (10.8)		8 (9.4)		35 (8.4)	
	Other	142 (78.8)		125 (85.0)		71 (83.5)		338 (82.0)	
	No response	27 (14.8)		6 (4.2)		6 (7.1)		39 (9.6)	

Race, gender, and undergraduate major of the participants were obtained from the Office of Curriculum and Scholarship internal records, which were optionally self-reported by students. No response indicates the percentage of students who did not opt to self-report their information.

### Participation in the HIMI

**Figure 2:**
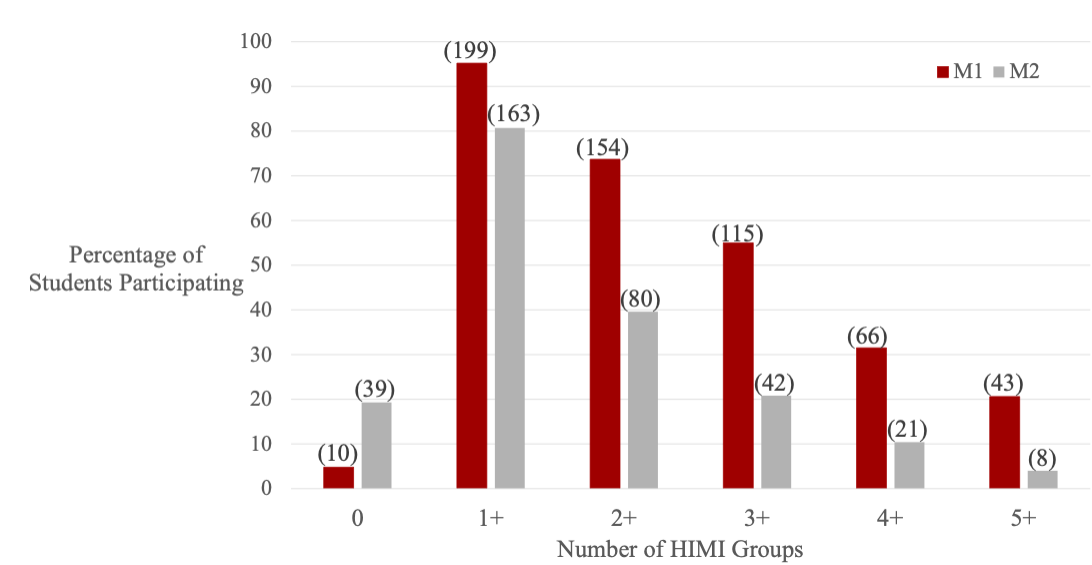
Medical student participation in the Humanism in Medicine Initiative (HIMI) groups, displayed by minimum participation levels. Each HIMI group includes all students who participated in the minimum amount stated or more. Medical student participation in HIMI groups was defined as attending any event hosted by a student group in the HIMI, organizing an event, leading a group or event, and nominating a peer for a nomination-based group between August 2018 and July 2019 and collected from internal HIMI records.

First- and second-year students vastly participated in HIMI student groups. The HIMI participation rate was 95% (n=199 students) among first-year students and 81% (n=163) among second-year students, accounting for a total participation rate of 87% (n=362). First-year students averaged participating in 3.0 unique student groups, and second-year in 1.6 unique student groups (Figure 2). Of the HIMI groups, Peer Mentoring (n=333), Somali Health Initiative for Nutrition Education (n=62), Visual Arts (n=49), M1 Fall Retreat (n=43), and Dance in Medicine (n=39) were those most participated in.

Student participation was categorized as low (0-1 groups), medium (2-3 groups), or high (≥4 groups). When compared with the total study population, high participation was observed to a larger extent among Black or Asian (Black: n=35, 8.5% vs n=8, 9.4%; Asian: n=82, 19.9% vs 22, 25.8%), among female participants (n=188, 45.4% vs n=54, 63.5%) and those who have majored in humanities during their undergraduate studies (n=35, 8.4% vs n=8, 9.4%).

### Academic Outcome Results

Step 1 USMLE scores were analyzed for second-year students. HIMI participation had no significant relationship with the OSCE outcomes. When comparing low and high participation, the high participation group averaged higher scores (238.0, SD 14.6) than the low participation group (233.7, SD 19.3) (*P*=.10), though the difference was not significant.

### Stress and Burnout

Perceived stress was found to be a significant factor by participation level (*F*_2,193_=3.85; *P*<.05; η_p_^2^=0.038). A post hoc analysis was conducted given the significance of the omnibus test. The Tukey honestly significant difference multiple comparison test was used to examine all pairwise relationships. A significant difference was found between the students with medium participation (18.5, SD 6.6) and students with low participation (15.7, SD 5.58; *P*=.02). This finding indicates that medium participation in the HIMI was significantly associated with higher levels of perceived stress than those with low participation in the HIMI. Participation in the HIMI was not significantly associated with burnout, sense of belonging, or QOL.

### GHHS Induction

GHHS induction was significantly associated with participation in a HIMI service (*χ*^2^_1_=5.8, *P*<.05) and leadership focused group (*χ*^2^_1_=19.3, *P*<.001).

## Discussion

### Principal Findings

This study investigated the short-term impact of first- and second-year students’ participation in the HIMI on their stress, burnout, and academic success. We found that the HIMI is utilized by the majority of first- and second-year students (95% vs 81% participation, respectively), and those who participated in ≥4 HIMI groups were more often female, Black, Asian, or had a humanities undergraduate major compared to the total study population.

Further, students who participated in a service- or leadership-focused group were significantly more likely to be inducted into the GHHS than those who did not participate in these groups.

Medium participation (defined as participation in 2-3 HIMI groups), compared with low participation (0-1 groups), was associated with significantly higher stress. Medical student stress and burnout have been a concern in recent decades with no gold-standard solution described. The association of medium HIMI participation with high stress warrants future study at our institution, with more medical students, and at other institutions with similar programs. Perhaps students with higher baseline stress seek out HIMI for reduction, but it is difficult to determine the cause of stress, as a complex multidimensional variable, and the results of this study cannot infer causation. Furthermore, participation in the HIMI had no significant impact on Step 1 USMLE scores.

### Comparison With Prior Work

Humanities content during undergraduate medical education has also been a topic of debate for decades. Most recent concerns seek to determine how to balance the need for humanistic training and diverting students’ time from rigorous basic science study to accomplish this. Moreover, the success of humanities content as part of the curriculum versus as an extracurricular program has similarly been debated. Our results suggest that students highly utilize the HIMI as a voluntary opportunity to engage with the humanities. This study found first-year students to engage with HIMI to a greater degree than second-year students, possibly owing to the increased demands on second-year students to study for the Step 1 USMLE. HIMI membership was also more commonly observed among students who are female, Black, Asian and had a humanities background than the overall medical school population. One possible reason for this result could be that students with an identity underrepresented in medicine (female, Black, or Asian students and those with a humanities education) sought out the HIMI as a community of support.

The association of HIMI leadership and service engagement with GHHS membership suggests that participation in the HIMI makes students more competitive for GHHS membership, possibly either by characterizing time spent on valued activities or identification of intrinsic interest in humanities. While the GHHS and HIMI appear to share several values, the GHHS selection process requires strong peer nominations as the first step for consideration. HIMI involvement may therefore add holistic review of applicants.

However, students’ time spent in the HIMI did not significantly impact Step 1 USMLE scores in our study, and these findings may be evidence for including humanities content in the medical students’ schedule without concern for detracting from basic or clinical science education.

### Limitations

This study was completed at a single medical school in an academic institution with a small sample size of students, compared to the population of medical students in the United States. The results of the study are therefore limited in generalizability within the United States and internationally. The study constituted a secondary data analysis and was limited in analysis by existing data. Further variables could be analyzed in future, experimentally designed studies. Participation data were collected internally by students and may be limited by program design. Further, participation was classified binarily and thus did not discriminate difference in volume of participation within each group. Some student group participation may be underrepresented in the study owing to these limitations. Student demographic and some outcome data were optional to report, which may have led to bias in our results. The analysis had a cross-sectional design and did not account for longitudinal outcomes of participation in the HIMI. The study utilized solely quantitative data, which may limit the ability to capture the full impact of humanism on medical students [46]. Finally, the study was observational by design.

### Conclusions

The HIMI is an extracurricular program vastly utilized by first- and second-year medical students at OSUCOM and did not impact Step 1 USMLE scores. Medium participation in the HIMI was associated with higher stress, and service- and leadership-focused HIMI participation was associated with a higher level of induction to the GHHS. This study identified areas for future studies to understand the association of participation in the HIMI with stress, academic success, potentially important activities for social support and resilience in medical school.

## Abbreviations

GHHS: Gold Humanism Honors Society
HIMI: Humanism in Medicine Initiative
MBI: Maslach Burnout Inventory
OSCE: Objective Structured Clinical Examination
OSUCOM: Ohio State University College of Medicine
PCS: Perceived Cohesion Scale
PSS: Perceived Stress Scale
QOL: quality of life
USMLE: United States Medical Licensing Examination
